# Molt-related changes in the major ampullate silk gland of the barn spider *Araneus cavaticus*

**DOI:** 10.1080/19768354.2020.1837950

**Published:** 2020-10-29

**Authors:** Myung-Jin Moon, Edward K. Tillinghast

**Affiliations:** a Department of Biological Sciences, Dankook University, Cheonan, Korea; b Department of Biological Sciences, University of New Hampshire, Durham, NH, USA

**Keywords:** Ampullate gland, *Araneus cavaticus*, fine structure, molt, spider silk

## Abstract

Spiders molt periodically before reaching full maturity, but several spiders continue to molt after sexual maturity. This post-maturity molting (PMM) behavior has been observed in the barn spider *Araneus cavaticus* (Araneae: Araneidae) among the orb-web spiders. In this study, we investigated molt-related changes in the ampulla and tail regions of the major ampullate gland during the PMM sequences (intermolt, pre-molt, ecdysis, and post-molt). The results showed that all gland units consist of a monolayer of epithelial cells surrounding a large central lumen, and two types of secretory granules (Type-M and Type-S). During the molting period, most cells showed fine structural modification in their organelles, and conspicuous tissue swelling was detected at the glandular epithelium. Following the molting cycle, the amount of Type-M granules continues to increase in the cell with a corresponding swelling, but Type-S granules gradually disappeared during the process of ecdysis. This suggests that the molt-related changes in spider silk production originates from the periodic production of Type-S secretory granules in the ampulla region. As Type-M granules flow toward the funnel, it is coated with viscous liquid secretion of Type-S granules in order to produce dragline silk fibers. We provide fine structural evidence for Type-S granules of hexagonal crystalline substructures representing glycoprotein substances to maintain high level of water content.

## Introduction

Molting, also known as ecdysis in many invertebrates, is a natural process by which animals routinely casts off parts of body at certain points in their life cycle (Nation 2015). In particular, molting in arthropods can include shedding of the entire exoskeleton (Nijhout 2013). The main function of the repeated shedding of the arthropod integument is to enable morphogenesis and more extensive growth of the body (Cheong et al. 2015).

In arthropods, molting normally stops when sexual maturity is achieved, but some species continue to molt after sexual maturity. This post-maturity molting (PMM) behavior has been observed in insects (Christiansen 1964) and in many species of lobster, crab and shrimp (Nijhout 2013). In spiders, the occurrence of PMM has also been reported in black widow spider *Latrodectus mactans*, brown huntsman spider *Heteropoda venatoria*, orb-web spider *Nephila pilipes*, and tarantulas (Cheng et al. 2017).

Like other arthropods, spiders have an exoskeleton that they have to periodically shed as they grow (Foelix 2011). Since spiders stop feeding during the molt, such physiological condition directly affects the interaction between molting, including web building behavior as well as silk composition. The developmental stages of arthropods are subdivided into a series of instars through repeated molting. Each instar has a degree of morphogenesis and other characteristics that are unique to certain species (Cheong et al. 2015). The number of molts in spiders varies for both species and gender but is generally between 5 and 9 times before sexually matured (Foelix 2011).

The molt cycle of spiders encompasses the period between two successive molts and has been subdivided into 4 major stages; intermolt, pre-molt, molt (ecdysis), and post-molt (Kuballa et al. 2011). Most spiders molt throughout their lives, and periodic replacement of cuticle is intrinsically linked with their physiology (Cheng et al. 2017). In spiders, molting is thought to be triggered and controlled by hormones (Cheng et al. 2017), as increased levels of ecdysone in the hemolymph of spiders were found several days prior to ecdysis (Bonaric and De Reggi 1977). Physiological processes that release old exoskeleton from underlying tissues typically cause various structural changes (Higgins 1990) which are without exception consistent with spider silk production system.

Production of silk substances within the glandular epithelium of the silk gland has been intensively studied (Akai 1971; Tillinghast and Townley 1986; Kovoor 1987; Moon 1998; Moon et al. 1998; Townley et al. 2006). The secretory silk substance of the major ampullate gland is known to originate from the rough ER of epithelial cells, and they are grown by fusion with surrounding small vesicles to form the secretory granules (Moon and Tillinghast 2004). In particular, molt-related changes in the morphology of the ampullate spigots on the spinneret and related internal silk glands have been reported in both of the barn spider *Araneus cavaticus* (Townley et al. 1993) and the garden spider *Argiope bruennichi* (Moon 2012).

However, information on histological changes in the silk gland associated with molt during spider silk production is limited (Moon and Tillinghast 2002, 2004). In the major ampullate gland, the synthesis of each type of secretory granule is not always uniform over the length of the gland (Kovoor and Peters 1988; Moon et al. 1998). Previously, the synthesis of silk material by the major ampullate glands could be studied by either cholinergic stimulation of the nervous system (Peakall 1964) or by depletion of the silk reservoir by mechanical pulling stimulation (Tillinghast and Townley 1986; Moon and Tillinghast 2004).

PMM is considered to be one of the key mechanism of extreme sexual dimorphism in arthropods (Kuntner et al. 2012). Although molting is triggered by hormones and environmental factors (Cheong et al. 2015), PMM has received little attention so far. Therefore, this study was designed to examine the processes of silk gland remodeling during the post-maturity molting (PMM) of orb-web weaving spiders. In particular, the fine structural examination of the silk glands and their molt-related changes have been nearly neglected except for physiological studies involving some morphological observations (Higgins 1990; Vetter and Rust 2010; Kuntner et al. 2012; Cheng et al. 2017; Vetter et al. 2017). We describe the histologic and fine structural examination of the glandular epithelium in the major ampullate silk gland with aid of light and high-resolution biological transmission electron microscopes.

## Materials and methods

The barn spiders, *Araneus cavaticus* were collected during winter season and reared in rectangular cages in the laboratory greenhouse of University of New Hampshire, Durham NH, USA. All spiders were maintained under ambient conditions with natural lighting in enclosures comprising a wooden frame (height X length X width = 50 X 50 X 10 cm) with glass panels on the front and back, and fed house flies (*Musca domestica*) and water daily.

A total of 16 adult PMM spiders (4 spiders per 4 stages of molting) of both male and female individuals were used for this experiments. To select exact timing of molting, sample selection techniques described by Townley et al. (1993) were used. Spiders that make orb-webs and actively feed on houseflies have been classified as intermolt stage. However, spiders that cease web construction and feeding have been classified as pre-molt stage, since they basically start ecdysis within 3 days. Following ecdysis, the spiders that started building the first orb-web were classified as post-molt stage.

For histologic preparation, specimens were anesthetized with CO_2_ and dissected under a dissecting microscope in a drop of spider Ringer's solution consisting of 160 mM NaCl, 7.5 mM KCl, 4 mM CaCl_2_, 1 mM MgCl_2_, 4 mM NaHCO_3_, 20 mM glucose, pH 7.4 (Moon and Tillinghast 2013). Among the various silk glands in the abdominal cavity, the major ampullate silk glands were gently removed and fixed in alcoholic Bouin's solution. After fixation, the specimens were dehydrated through an ethanol series from 30 to l00% (30 min at each concentration with one repeat at 100% ethanol), and transferred to xylene for clearing at room temperature to 60℃. Subsequently, they were embedded with Paraplast embedding medium (Fisher Scientific Co., Pittsburgh, Pa, USA) at 60℃. The sections were cut with a thickness of approximately 5 µm using a rotary microtome, Histocut 820-II (Reichert-Jung, Germany) and they were stained with hematoxylin and eosin (H and E) solutions.

For transmission electron microscopic (TEM) experiment, the major ampullate silk glands were gently removed and fixed in a mixture of 2% paraformaldehyde and 2.5% glutaraldehyde buffered with cacodylate buffer at pH 7.4. Post-fixation was performed with 1% osmium tetroxide (OsO_4_) and washed several times in the same buffer solution following fixation. The specimens were then dehydrated in ascending concentrations of ethanol and embedded in Poly/Bed 812-Araldite medium (Polysciences Inc., Warrington, PA, USA) via propylene oxide (Moon 2018).

Semi-thin sections with 0.5-1.0 µm thick were obtained using a LKB Ultratome V (LKB, Stockholm, Sweden), and they were stained with 1% toluidine blue (dissolved in 1% borax). They were photographed using Zeiss Axiophot microscope (Carl Zeiss, Jena, Germany) coupled with Motic digital imaging system (Motic Instruments Inc., Richmond, Canada). Ultrathin sections were obtained using an Ultra 45° diamond knife (Diatome, Hartfield, PA, USA), and were double stained with uranyl acetate, followed by lead citrate. After these treatments, the sections were examined with a JEM 100 CX-II transmission electron microscope (JEOL Ltd., Tokyo, Japan) at 80 kV.

## Results

**Figure 1.**
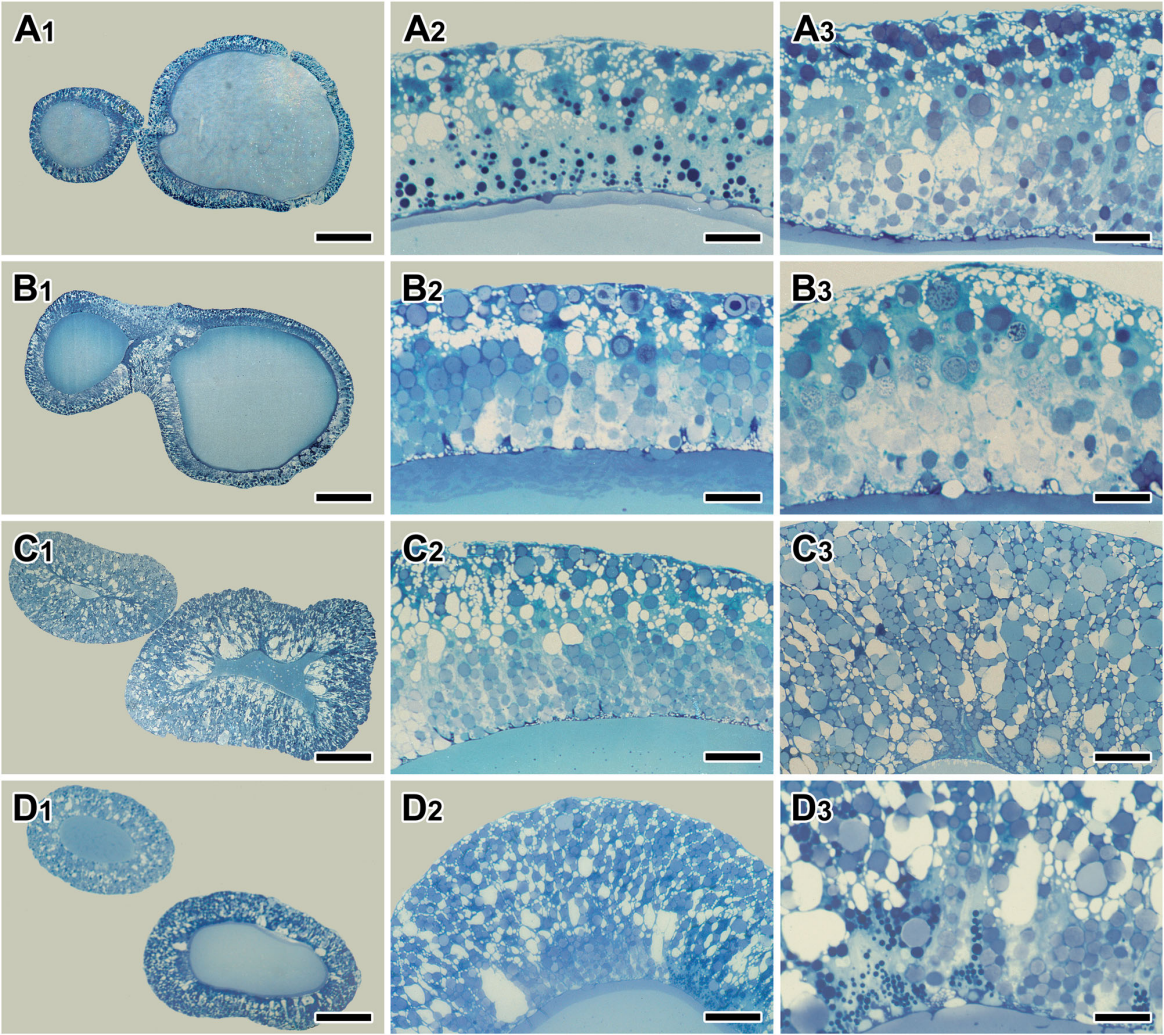
Photo micrographs of the major ampullate gland during the post maturity molt sequences – intermolt (**A_1_-A_3_**), pre-molt (**B_1_-B_3_**), ecdysis (**C_1_-C_3_**), and post-molt (**D_1_-D_3_**). At the intermolt and post-molt period, two types of secretory granules are distributed throughout the epithelium. However, only one type of secretory granules and severe swelling of epithelial tissue appeared at the pre-molt and molt period. Scale bars indicate 100 µm (A_1_, B_1_, C_1_, D_1_), 25 µm (A_2_, B_2_, C_2_, D_2_), and 10 µm (A_3_, B_3_, C_3_, D_3_), respectively.

The major ampullate gland is the most prominent silk gland with the largest volume in the barn spider *A. cavaticus*. Each major ampullate gland consists of three functional regions; duct, ampulla and tail. To observe the fine structural modification in silk-producing organs, both the ampulla and tail regions of the major ampullate gland are examined. In particular, molt-related changes in the major ampullate silk gland were analyzed following the 4 stages of molting cycle; intermolt (Figure 1(A_1_-A_3_)), pre-molt (Figure 1(B_1_-B_3_)), molt or ecdysis (Figure 1(C1-C_3_)), and post-molt (Figure 1(D_1_-D_3_)).

During the intermolt period, simple columnar epithelium lines ampulla region of the major ampullate gland. In tissue sections stained with toluidine blue, two types of secretory granules are distributed evenly throughout the area (Figure 1(A1-A_3_)). During the pre-molt period, one type of secretory granule is gradually disappeared, and another type of granule is very prominent because the compact granules are stained blue (Figure 1(B1-B3)). During the molting stage, on the other hands, gland swelling of the ampulla region is the most common histological modification (Figure 1(C_1_-C_3_)). This histological modification is particularly remarkable both stages of molting and post molting (Figure 1(D_1_-D_3_)).

**Figure 2.**
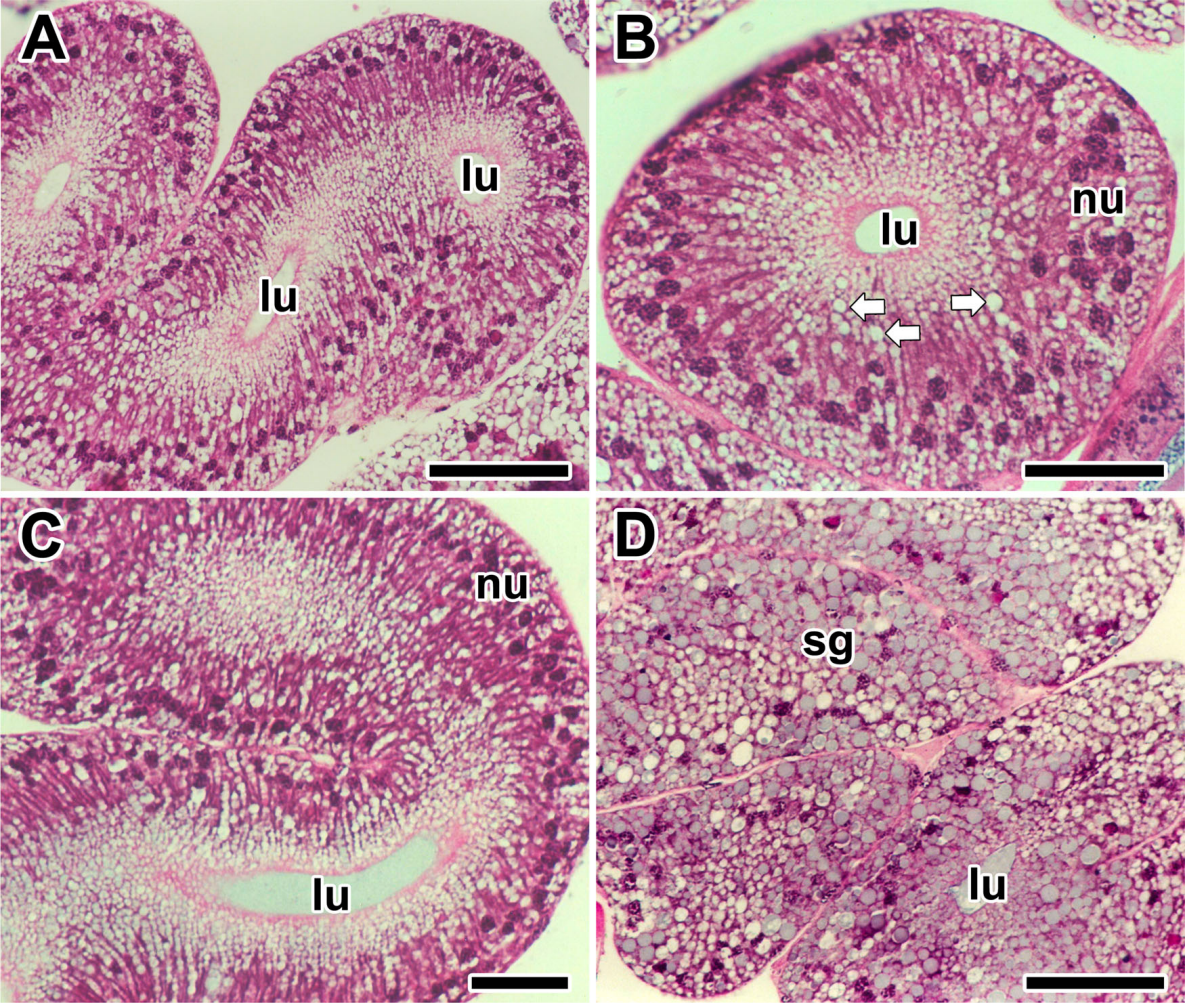
Photo micrographs of the major ampullate silk glands stained with Hematoxylin and Eosin at the intermolt stage of the spider *A. cavaticus*. **A, B:** Glandular epithelium is composed of columnar epithelial cells and filled with numerous secretory granules (arrows). **C, D:** Surface of the epithelium highly protrudes toward the lumen (lu). Secretory granules (sg) are accumulated at apical region of the cells. nu: nucleus. Scale bars indicate 10 µm (A, C) and 5 µm (B, D), respectively.

The appearance of the glandular epithelium is somewhat variable in different experiments even though identical staining methods were used. However, tissue sections stained with Hematoxylin and Eosin show that most of the epithelial cells are filled with numerous secretory granules, and convoluted secretory regions of the major ampullate gland looks like thick and swollen tubular pouches during the intermolt period (Figure 2(A)).

A single type of secretory cell constitutes the secretory area, and the wall is composed of columnar epithelial cells (Figure 2(B)). Observation of the major ampullate gland is limited to the secretory area where transient secretory granules occur. However, droplets are frequently seen being stored in the apical area of the cells (Figure 2(C)). Particularly, increase of glandular epithelial cell clusters by an external volume expansion is remarkable. The secretory granules are actively produced within the glandular epithelium (Figure 2(D)).

**Figure 3.**
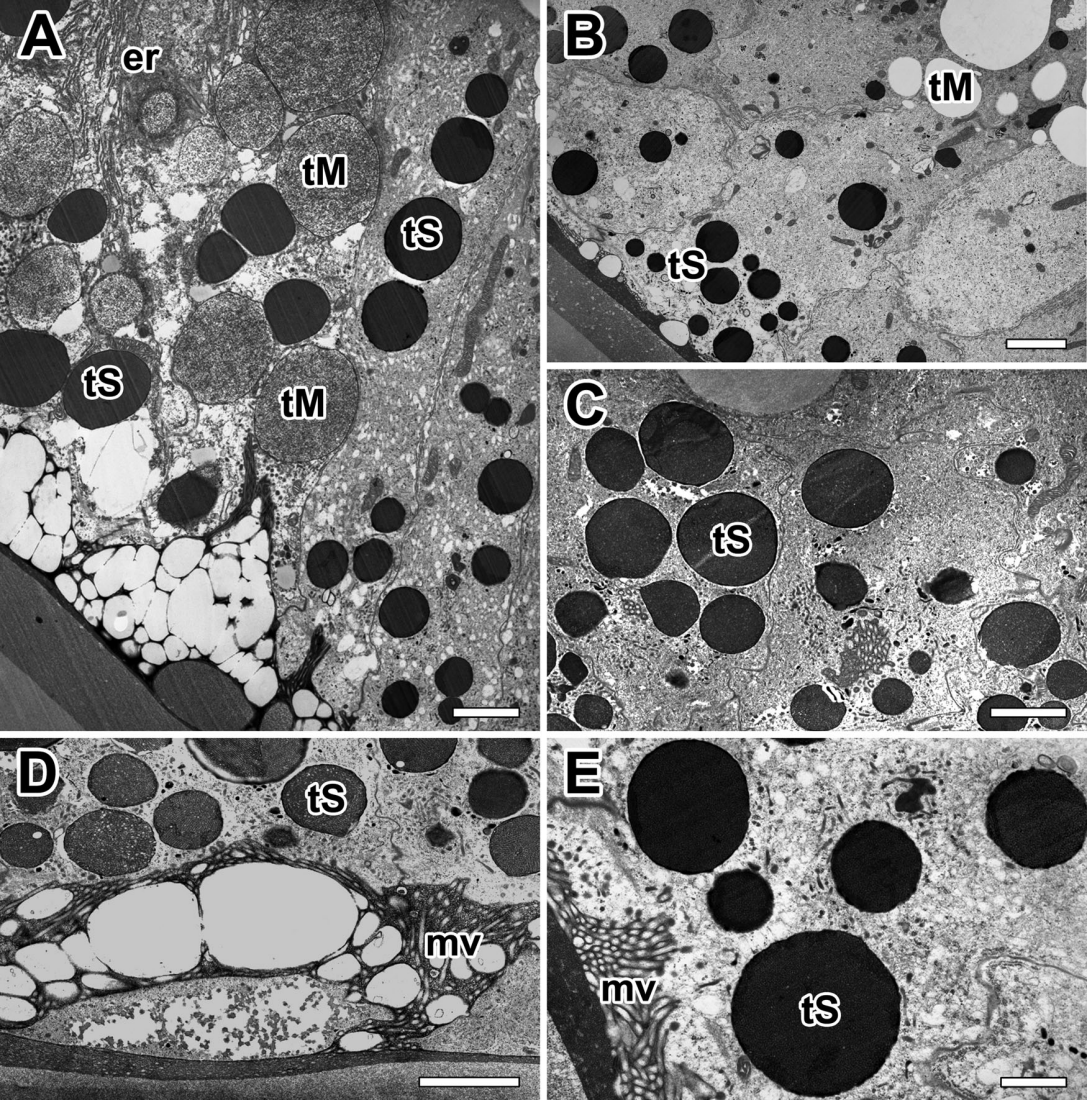
Electron micrographs of the major ampullate gland in *A. cavaticus* during intermolt period. **A, B:** Two types of secretory granules appeared. Type-S granules (tS) are seen at the apical region and Type-M granules (tM) are near the base of epithelial cells. **C:** Type-S granules which have electron-dense fine granular appearance are seen during the intermolt period. **D:** As Type-M granules flows toward the funnel, it is coated with secretion of Type-S granules. **E:** Type-S granules have a similar structure regardless of size. er: rough endoplasmic reticulum, mv: microvilli. Scale bars = 0.5 µm (A, B) and 0.2 µm (C-E), respectively.

On the basis of their morphology and histochemical characteristics, we carried out to investigate fine structural changes in the glandular epithelial cells of the major ampullate glands. Basically, two types of secretory granules occur in the major ampullate gland: one type (Type-M granules) is basically found in the base of secretory epithelium and the other (Type-S granules) near the apical region. The basal region of the cells is occupied by nucleus, a large amount of rough ER and mitochondria. The rest of the cell is filled with those of Type-M granules which are mucous type. While on the other, Type-S granules of the major ampullate gland are serous type (Figure 3(A,B)).

In electron microscopy, cells with Type-S granules are bound by a distinct membrane and appear to have an electron-dense fine granular appearance. Such granules are easily seen in the major ampullate gland cells of intermolt period where active silk production occurred (Figure 3(C)). As Type-M granules flow toward the funnel, it is coated with secretion of Type-S granules in order to produce final dragline silk fibers (Figure 3(D)). The granules of small size near the rough Endoplasmic Reticulum are homogeneously dense, however, the Type-S granules have a similar structure regardless of their size (Figure 3(E)).

**Figure 4.**
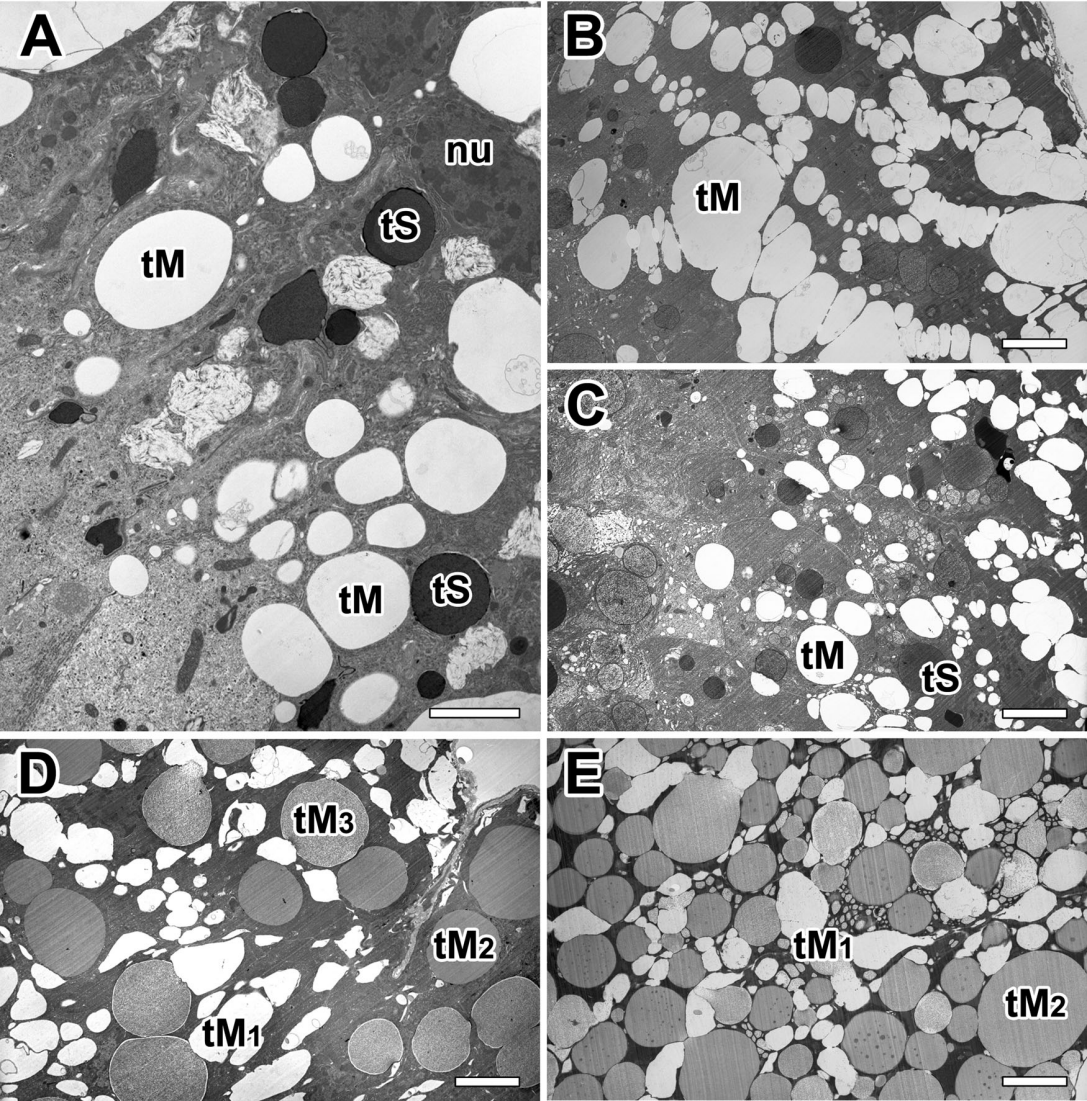
Electron micrographs of the major ampullate gland in *A. cavaticus* during pre-molt period. **A:** Glandular epithelium contains two types of secretory granules with varying sizes and densities. **B, C:** Electron lucent secretory granules accumulated at the basal region appear almost spherical vesicles. Large vesicles are formed by fusion with small vesicles from rER. (D,E) Type-M granules (tM) are composed of homogeneous internal materials with varying electron densities. Electron densities are changed from electron-lucent (tM1) to membranous (tM2), and finally fine fibrous appearance (tM3). nu: nucleus, tS: Type-S granule. Scale bar = 1 µm (A) and 0.5 µm (B-E).

In *A. cavaticus*, stopping movement to conserve energy and refuse food is typically a good sign of pre-molt. At the pre-molt stage, the spider is preparing ecdysis for shedding of the exoskeleton. The secretory epithelium of the gland also has two distinct types of secretory granules (Figure 4(A)). Both types of granules consist of a single type of tall columnar cells filled with separate secretory granules with varying sizes and densities. In contrast to the intermolt stage, the secretory epithelium is progressively changed to thicker cells with less definitive cell membranes (Figure 4(B)).

There is relatively small amount of Type-S granules, thus causing the epithelium to stain much lighter than the intermolt stage (Figure 4(C)). It contains abundant Type-M granules in their cytoplasm, and the granules are composed of homogeneous internal materials with distinctly different densities (Figure 4(D)). Regardless of the size and density of the granules, each granule contains an electron-lucent material with a fine fibrous appearance. This material appears to be in two phases with different densities in many granules. Following the maturation state of granules using the granule morphology, the mature granules were found to have a darker stained appearance (Figure 4(E,F)).

**Figure 5.**
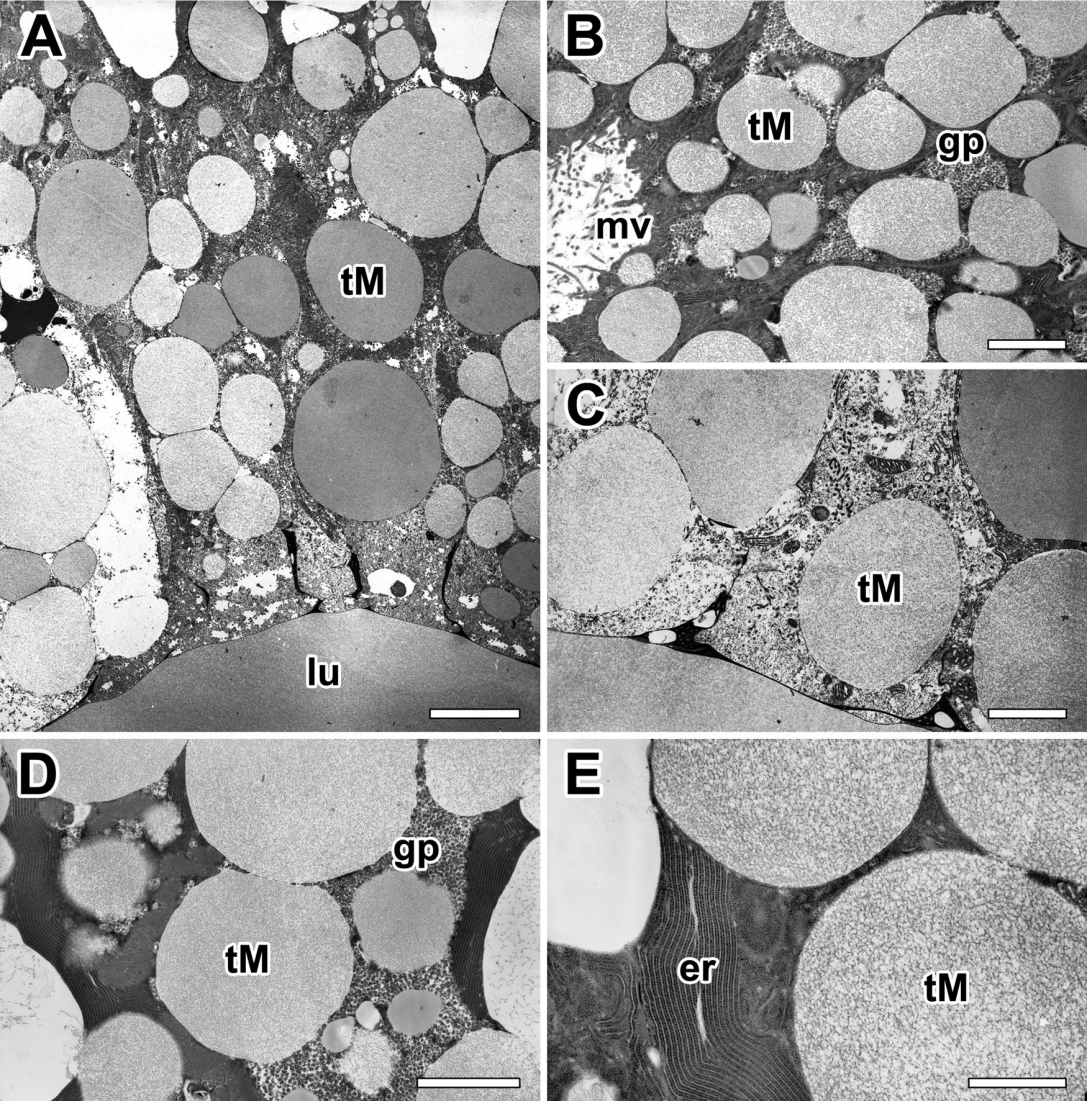
Electron micrographs of the major ampullate gland in *A. cavaticus* during the molting period. (A) Due to epithelial swelling, thickness of glandular epithelium is profoundly enlarged. (B) Cells are filled with Type-M secretory granules (tM), but Type-S granules are not observed. (C) Type-M secretory granules show a spherical appearance with low electron density commonly. (D, E) Precursors of the Type-M granules are produced as separated vesicles via well-oriented membranous cisternae of rER, but no Golgi complex has been found. gp: glycogen particles, lu: lumen, mv: microvilli. Scale bars indicate 1 µm (A), 0.2 µm (B-D), and 0.1 µm (E). respectively.

The major ampullate glands of the molting period present a different appearance. The thickness of glandular epithelium is profoundly enlarged, and conspicuous glandular swelling was detected. At molting period, whole cells are compactly filled up with secretory granules (Figure 5(A)). Type-S granules eventually disappear, and Type-M granules are the only secretory granules of the major ampullate gland at molting stage. The amount of Type-M secretory granules continues to increase in the cell with a corresponding swelling of the glandular epithelium (Figure 5(B)). They are spherical appearance and show a low electron density similar to those of mucous secreting cells. Within each granule, fine fibrillar structures occur (Figure 5(C)).

Granules containing more condensed fibrillar structure are also found in the gland at the late stage of the molt. However, granules containing denser matrix appear to be more abundant in the gland of the post-molt period. Most cells showed fine structural modification in their organelles, and highly organized substructures are found in the dense matrix of these granules (Figure 5(D)). Rough ER (rER) is found throughout the cell but the density is higher near the nucleus. High magnification electron micrograph reveals that the secretory precursors of the major ampullate gland are produced as separated vesicles via well-oriented membranous cisternae of rER, and no Golgi complex has been found in these cells (Figure 5(E)).

**Figure 6.**
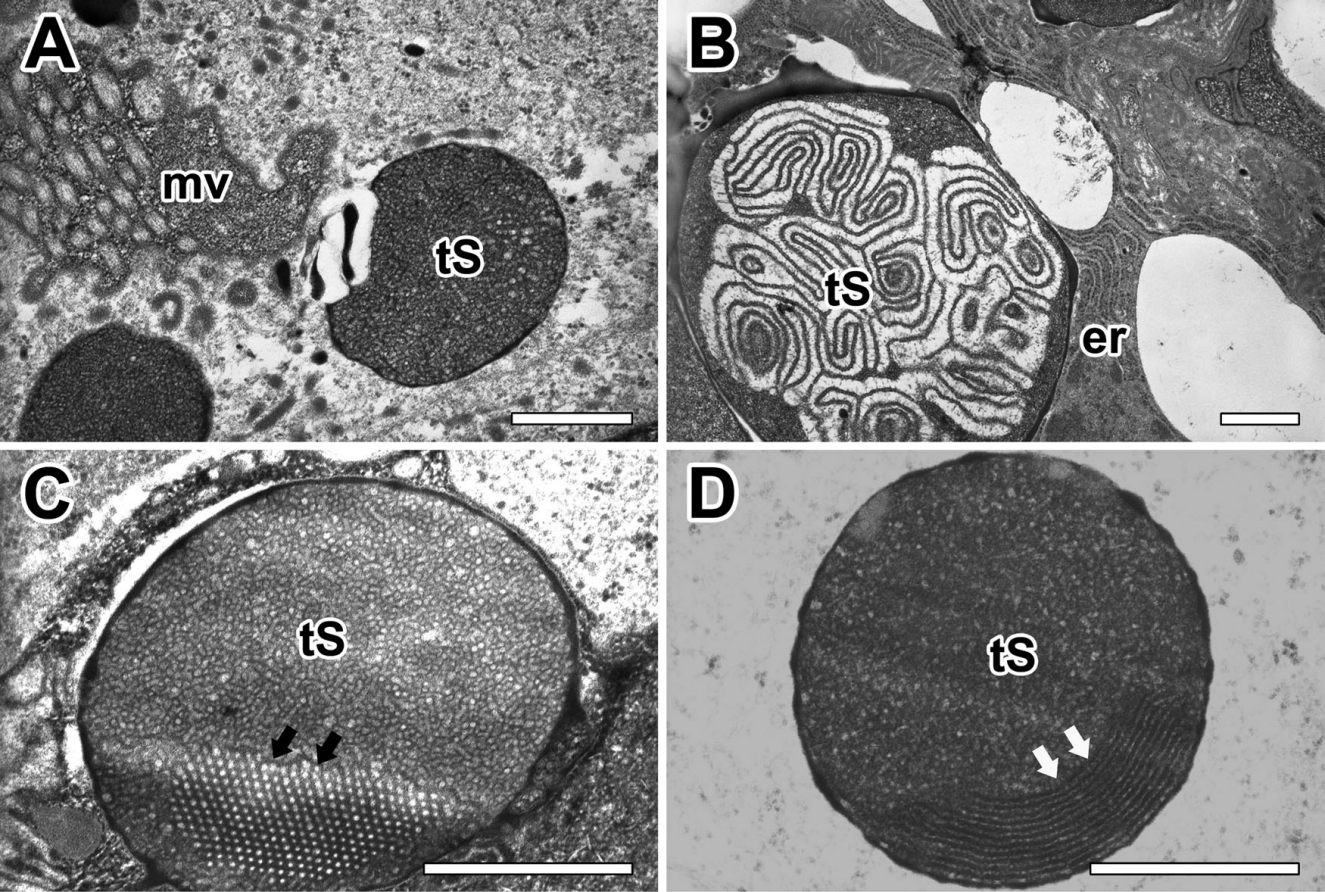
Electron micrographs of secretory granules in the major ampullate gland. (A) Type-S granules (tS) are characterized as spherical vesicles with electron-dense internal material delimited by a typical membrane. (B) Mature granules show electron-dense fine granular core and an outer crystalloid substructure. (C,D) Some granules which have a honeycomb-like (black arrows) or periodic crystalloids lamellae (white arrows) structures are believed to represent the crystalline part of the granules. er: rough endoplasmic reticulum, mv: microvilli. All scale bar indicates 0.1 µm.

Both secretory granules of the major ampullate gland were commonly synthesized from rER of glandular epithelial cells. Highly-developed cisternae of the rER surrounded these granules in basal region, however Golgi complex does not seem to play an important role in the process of secretion (Figure 6(A)). The mature secretory product of Type-S granule appears almost spherical vacuoles with no more than 0.5 µm in diameter. They are characterized as electron-dense structures with very dense amorphous material delimited by a typical membrane. Small lamellar-shaped inclusions were present in some of these granules (Figure 6(B)). This distinctive granular morphology is found only in Type-S granules. Mature granules can show electron-dense fine granular core and an outer crystalloid substructure all bounded by a typical membrane. A few granules appeared as a honeycomb-like structure (Figure 6(C)). The periodic structure of the crystalloids became apparent at high magnification. The more resistant lamellae are believed to represent the crystalline part of the granule (Figure 6(D)).

**Figure 7.**
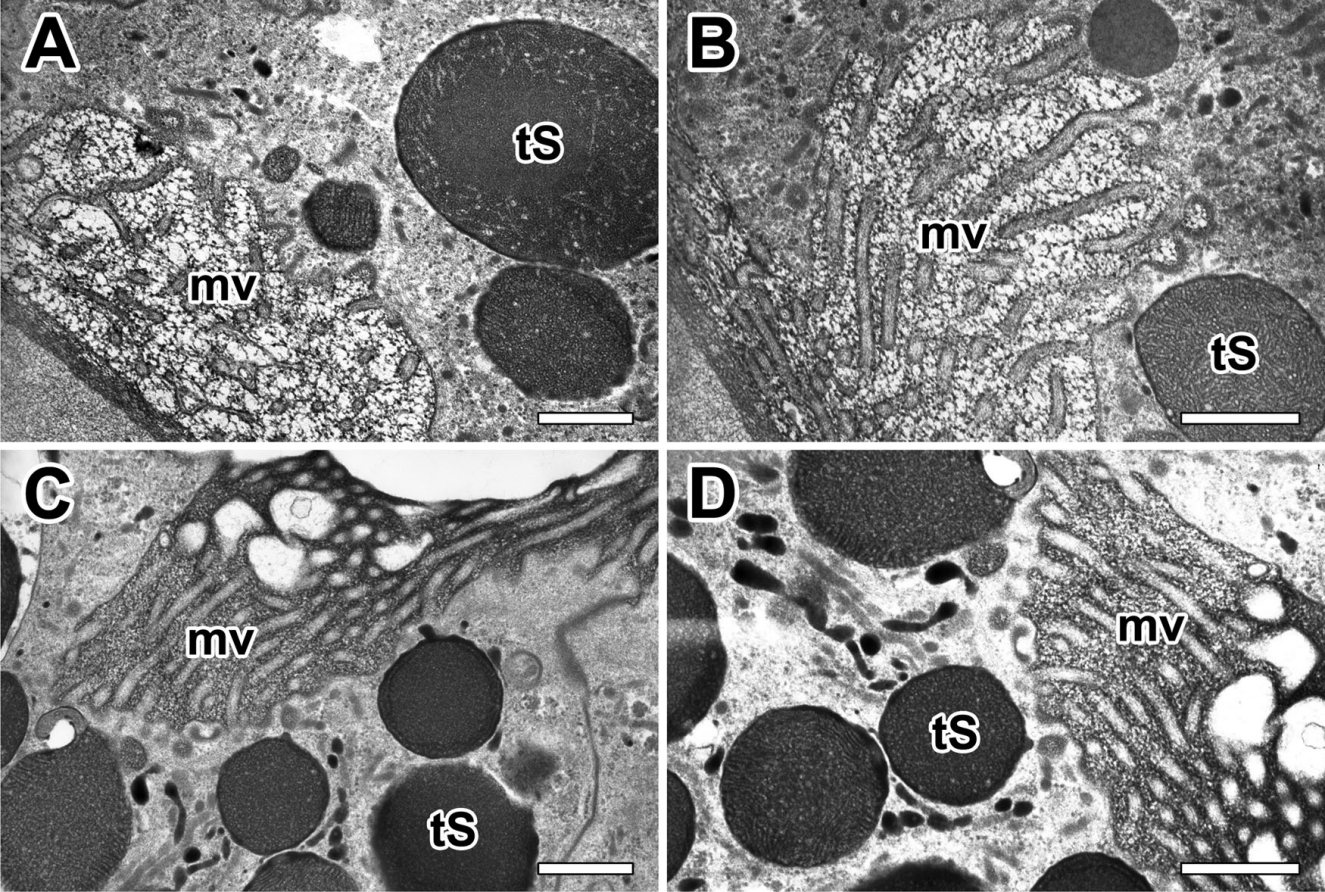
Electron micrographs of typical appearance of microvilli at apical epithelial cells in the ampullate gland. A: Brush border of an epithelial cell shows tightly packed microvilli (mv) that extend to lumen. B, C: Without granular fusion, the Type-S granules (tS) are finally released through the brush border by mechanism of merocrine secretion. D: Final secretory granules at luminal cytoplasm can be seen as a form of electron-dense granules. All scale bar indicates 0.1 µm.

Our TEM micrographs depict the typical appearance of microvilli on the apical surface of the epithelial cells with a brush border. The fine-structure of the apical surface of the major ampullate gland was in most cases seen as short projections of microvilli measuring less than 0.3 µm in length (Figure 7(A)). Each granule characterized by progressive emptying of the secretory granules without granular fusions and specific release of granule contents (Figure 7(B)). Type-S granules are finally released across the brush border of microvilli by merocrine secretion (Figure 7(C)). Final secretory granules at luminal cytoplasm can be seen as a form of electron-dense granules. Apparently, the electron densities of these granules increase according to their maturational levels (Figure 7(D)).

## Discussion

Molting is a cyclic process that occurs in all arthropods and is essential for growth, reproduction and metamorphosis. Spiders, like other arthropod, also grow by molting. Most spiders have multiple silk glands and secrete different types of silk material that are optimized for different purposes (Foelix 2011). Several types of silk glands are remodeled during molting and may not be fully functional for a few days immediately after molting (Townley et al. 2006).

Since the barn spider *A. cavaticus* also undergoes a remodeling process during PMM, the major ampullate glands are an ideal storage location for crucial silk components for extensive dragline silk and allowing for rapid replenishment of the major ampullate silk following molting. In other words, spiders could produce excessive dragline silk from the major ampullate gland, and then use the silk amino acids to make new silk once the molting process is over. The highly efficient recycling of web parts thereby significantly reduces the costs of silk production by reusing related amino acids (Opell 1998).

Our histological experiments on glandular epithelium of the major ampullate silk gland in the barn spider *A. cavaticus* show a characteristic tissue swelling response as a consequence of molt-related changes. In particular, the luminal surface of the epithelium is highly protruded toward the lumen, and cells are filled with numerous secretory granules during the molt. The reason for the tissue swelling in the silk gland of spider during the molt is unclear. However, following two arguments support the premise that the swelling of the tissue we identified for function is a very important histological evidence of the modifications associated with major ampullate silk gland molting.

First, the physiological process of molting typically causes various structural changes with the spider silk production system (Moon and Tillinghast 2002, 2004). If a spider is able to molt successfully without the risk of food storage, the adaptive effects of water and nutrient preservation may play a significant role (Hutchinson et al. 1997), as the spider stops feeding during the molting period. In particular, resource allocations directly affect the interaction between molting, silk composition, and web building behavior (Townley et al. 2006) since arthropod molting strategies are always combined with a loss of body mass and minimization of nutrient waste (Walter et al. 2008). It is therefore likely that spiders must swiftly resume their prey capture to compensate for lost foraging opportunities of the days after molting (Higgins 1990). Previous research has also shown that *Argiope* spider requires large amounts of wrapping silk that has to be newly synthesized after the molt for subsequent capture events (Higgins 1990).

Second, previous researches have shown that the molt itself, or ecdysis, involves the shedding of the exoskeleton through a rapid uptake of water from the environment, causing the exoskeleton to rupture (Akai 1971; Nation 2015). In addition, further water absorption during post-molt facilitates the expansion of a new soft exoskeleton, which is essential for animal growth (Vetter and Rust 2010; Nijhout 2013).

Current study clearly shows molt-related changes in the glandular epithelium and the fine structural modification of the secretory granules of the major ampullate gland. The results strengthen the premise that food shortage and preservation of silk substances during molting can serve as a major reason for tissue swelling. Therefore, this demonstrates that the molt-related changes in glandular epithelium are likely to be combined with the risk of food shortage (Townley et al. 2006; Cheng et al. 2017), and may be involved in preserving the integrity of the silk substances (Opell 1998; Crews and Opell 2006) during the molting period.

Additionally, blockage of the spinning tube may be the reason of tissue swelling in the spider. Feeding stimulates the production of silk material (Cheng et al. 2017), so when the spider stops feeding during ecdysis, the swelling of the glands worsens. If the spigot is blocked, the secretory silk material has no place to go and the gland swells, which is consistent with our histological observations. Spiders stop feeding to some extent not only during molting, but also before molting for several days (Vetter and Rust 2010). Since molting events are generally vulnerable phases in the life of the spider (Baba and Miyashita 2006) the protection of silk glands may be related to maintaining the integrity of the silk substances for web production during molting (Eisner and Nowicki 1983).

Our fine structural analysis of secretory granules reveals other characteristics of post maturity molting (PMM) affecting glandular silk production. The results showed that all gland units consist of single layer epithelial cells surrounding large lumens, and two types of secretory granules (Type-M and Type-S) are observed in the major ampullate gland. According to the classification of secretory granules with morphological characteristics in animal gland (Kim et al. 1970), it was found that these two types of granules definitely differ in substructures, such as density and size. The morphological appearance and relatively low electron density shown by Type-M granules in the ampulla and tail regions seem to be mucous type, while the Type-S granules in the ampulla region are serous type.

Previously, Kovoor and Peters (1988) first presented two types of secretory granules in the ampullate gland in *Polenecia producta*, demonstrating that the synthesis of each type of secretory granule does not occur in a uniform manner throughout the length of the gland. In addition, Vollrath and Knight (2001) observed that there are two distinct transverse zones in the secretory part of the major ampullate gland. They found that the spider silk gland not only synthesize silk protein themselves, but also store proteins that will function at the time of final assembly into fibrils. Moon and Tillinghast (2004) also reported that more than two types of secretory granules are closely related to the silk production of major ampullate silk gland.

Our electron microscopic examination of the two types of secretory granules reveals that the lower electron density granules with fine fibrous substructure may be classified ‘mucous-type (Type-M) granules’ as they resemble in appearance with the secretory granules of the mucous secreting cells (Kim et al. 1970). The granules, on the other hand, of higher density and distinct membrane are similar to the granules of granulated serous glands, so they are referred to as ‘serous-type (Type-S) granules’ (Kim et al. 1972). Both types of granules are localized at the apical region of the cytoplasm in each cells.

In cells containing Type-M granules, the cytoplasm is more abundant and a parallel profile of the endoplasmic reticulum is present throughout the cytoplasm. Type-M granules are membrane bound, but membranes are not distinct in many granules. In cells containing Type-S granules, segments of endoplasmic reticulum are mostly scattered at the basal region of the cytoplasm. The structure of the Type-M granules remains the same throughout the entire stages of the molting period. Type-S granules, on the other hand, show a variation in distribution between the molt and intermolt stages of the molting cycle.

Vollrath and Knight (2001) found that the secretory part of the major ampullate gland in orb-web spiders has two distinct zones, the A-zone and B-zone. As the secretion of A-zone flows towards the funnel, it is coated by a viscous homogeneous liquid secreted in the B-zone. The solution secreted by the A-zone is identified as spidroin (O'Brien et al. 1998) – the main proteins making up spider dragline silk. Whereas, the coat protein secreted in the B-zone is identified as glycoprotein in the thread biochemically (Weiskopf et al. 1996).

In *A. cavaticus*, Type-S granules show electron-dense fine granular core and an outer crystalloid substructure. At high magnification, some granules exhibit a honeycomb-like substructure. Previous TEM approach also detected the formation of honeycomb-like structures in secretory granules, and suggested them as structures with a continuous cross-linked network and abundant macropores (Janson et al. 1994). It is therefore likely that the secretory granules in the B-zone possess a high proportion of glycoproteins. This is consistent with our electron microscopic examination of the Type-S secretory granules which are filled with a hexagonal crystalline substructure.

Our TEM observation indicates that the Type-S granules are finally released into the lumen by merocrine secretion. It is then coated with secretions of Type-M granules which are likely to represent glycoproteins during silk dope production in the major ampullate gland. Previously, the secretion of the A-zone was reported to flow toward the funnel (Vollrath and Knight 2001), but it was not described as being coated with a homogeneous viscous liquid secreted from the B-zone. Since the glycoprotein may help to plasticize the thread by maintaining a high water content (Vollrath and Tillinghast 1991), the results of this study, together with previous studies, contribute biological and evolutionary knowledge of these molt-related changes during silk protein production. It seems that spiders have evolved over millions of year to preserve nutrient and water during molting period in order to overcome the risk of periodic shedding of their exoskeletons.
